# Heterologous Expression of Dehydration-Inducible *MfWRKY17* of *Myrothamnus Flabellifolia* Confers Drought and Salt Tolerance in *Arabidopsis*

**DOI:** 10.3390/ijms21134603

**Published:** 2020-06-29

**Authors:** Zhuo Huang, Han-Du Guo, Ling Liu, Si-Han Jin, Pei-Lei Zhu, Ya-Ping Zhang, Cai-Zhong Jiang

**Affiliations:** 1 College of Landscape Architecture, Sichuan Agricultural University, Wenjiang 611130, Sichuan, China; ghd199202@163.com (H.-D.G.); liuling@stu.sicau.edu.cn (L.L.); seraphimansuna@gmail.com (S.-H.J.); zhupeilei@stu.sicau.edu.cn (P.-L.Z.); 2 Department of Plant Sciences, University of California Davis, Davis, CA 95616, USA; zhyaping@ucdavis.edu (Y.-P.Z.); cjiang@ucdavis.edu (C.-Z.J.); 3 Crops Pathology and Genetics Research Unit, United States Department of Agriculture, Agricultural Research Service, Davis, CA 95616, USA

**Keywords:** *Myrothamnus flabellifolia*, resurrection plant, drought tolerance, gene function, molecular mechanism, WRKY transcription factor

## Abstract

As the only woody resurrection plant, *Myrothamnus flabellifolia* has a strong tolerance to drought and can survive long-term in a desiccated environment. However, the molecular mechanisms related to the stress tolerance of *M. flabellifolia* are largely unknown, and few tolerance-related genes previously identified had been functionally characterized. WRKYs are a group of unique and complex plant transcription factors, and have reported functions in diverse biological processes, especially in the regulation of abiotic stress tolerances, in various species. However, little is known about their roles in response to abiotic stresses in *M. flabellifolia*. In this study, we characterized a dehydration-inducible *WRKY* transcription factor gene, *MfWRKY17*, from *M. flabellifolia*. *MfWRKY17* shows high degree of homology with genes from *Vitis vinifera* and *Vitis pseudoreticulata,* belonging to group II of the WRKY family. Unlike known WRKY17s in other organisms acting as negative regulators in biotic or abiotic stress responses, overexpression of *MfWRKY17* in *Arabidopsis* significantly increased drought and salt tolerance. Further investigations indicated that *MfWRKY17* participated in increasing water retention, maintaining chlorophyll content, and regulating ABA biosynthesis and stress-related gene expression. These results suggest that MfWRKY17 possibly acts as a positive regulator of stress tolerance in the resurrection plant *M. flabellifolia*.

## 1. Introduction

Plants often suffer from multiple environmental stresses, such as pathogen infection, drought, higher levels of salinity, and extreme temperatures, which seriously affect the plant development as well as the final yield. With the change of global climate, the damage caused by abiotic stresses are about to increase in the near future [1]. To adapt to such environmental challenges, plants have evolved sophisticated defense mechanisms to regulate stress responses. Transcription factors (TFs) play essential roles in plant tolerance against biotic and abiotic stresses [2]. The regulatory functions and defense responses of a wide range of TF families have been elucidated, such as b-ZIP, NAC, MYB, and WRKY families [3].

WRKYs belong to a large and plant unique TF family [4]. The structure of all WRKY proteins includes the highly conserved amino acids WRKYGQK at its N-terminus and the zinc-finger-like motifs C-X4-5C-X22-23-H-X-H or C-X7-CX23-H-X-C at its C-terminus, which enable them to bind to the W-box in the promoter region of target genes with DNA sequence of (C/T)TGAC(C/T) [5]. Based on the amount of DNA binding and different zinc-finger-like motifs, WRKYs could be classified to three different groups: Group I features two WRKY domains comprising a conserved WRKY motif and zinc-finger-like motif; Group II is the largest group, which has one WRKY motif and one zinc-finger-like motif the same as that of group I (C-X_4–5_-C-X_22–23_-H-X_1_-H) (C_2_H_2_). This group was originally grouped into five different subgroups (IIa, IIb, IIc, IId, and IIe) [6]. Group III also has only one WRKY domain, but is distinguished from Group I and II by the zinc-finger-like motif showing as a C_2_-HC pattern (C-X_7_ -C-X23-H-X_1_-C).

Recent studies have shown that the WRKY family participates in a wide range of biological processes, including seed germination, plant development and phytohormone signalling [7]. One of the most critical functions of WRKYs is their involvements in defense against abiotic stresses [8,9]. In *Arabidopsis*, *AtWRKY1* participates in the stomatal closure to maintain moisture by regulating the membrane transporters [10,11]. The *AtWRKY63* knockout mutant has less tolerance to drought stress than the wild-type [12]. Overexpression of the *OsWRKY8* gene in *Arabidopsis* alters the morphology of root, thus enhancing drought tolerance [13], while overexpression of *GhWRKY34* in *Arabidopsis* shows a higher seed germination rate, cotyledon greening rate, root length, and chlorophyll content than wild-type plants under salt stress [14]. In addition, expression of the *GsWRKY20* gene in *Arabidopsis* significantly increased tolerance to cold stress [15].

*Myrothamnus flabellifolia*, as the most primitive angiosperm showing extreme tolerance to desiccation, is a woody homoiochlorophyllous resurrection plant distributing in the mountainous regions of Central and Southern Africa [16,17]. The molecular mechanisms underlying its tolerance to extreme drought and rapid recovery are still largely unknown. Using transcriptome analysis, Ma et al. (2015) found that many TFs (295) were responsive to dehydration [18]. The MYB, WRKY, and bHLH were among the largest groups during both dehydration and rehydration, among which nine *WRKY* genes were up-regulated in early dehydration. To understand the roles of *WRKY* genes in response to drought stress, we systemically attempted to functionally characterize these genes in a model plant, *Arabidopsis thaliana*, due to the technical challenge of transformation in *M. flabellifolia*. In this study, we reported functional studies of one of the dehydration-inducible WRKY TFs, MfWRKY17, which was named by its homology to group II of WRKY family member *AtWRKY17* (*At2G24750*) of *Arabidopsis* [18]. Our results show that overexpression of *MfWRKY17* in *Arabidopsis* enhanced drought and salt tolerances in transgenic plants, demonstrating that *MfWRKY17* plays important positive regulatory roles in response to abiotic stresses.

## 2. Results

### 2.1. Isolation and Sequence Analysis of MfWRKY17

**Figure 1:**
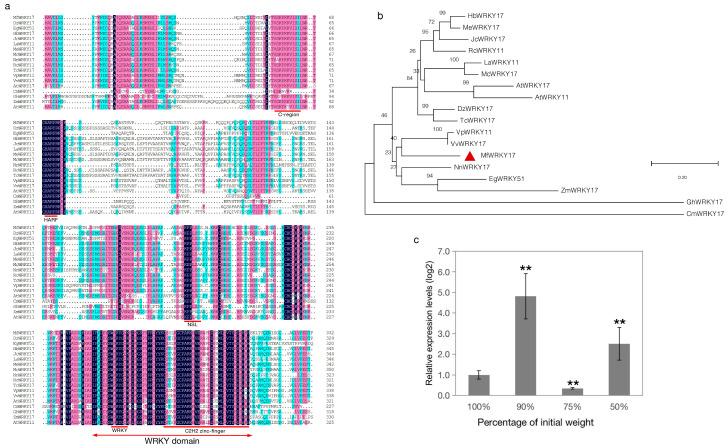
Sequence analysis and expression level of *MfWRKY17*. (**a**) Multiple alignment of deduced amino acid sequences; (**b**) Phylogenetic tree constructed using the neighbor-joining method, with the Jones–Taylor–Thornton model, Gamma Distribution, and 1000 Bootstrap replications. The details (sequence name: acceesion no., species) for sequences used were DzWRKY17: XP_022731890, *Durio zibethinus*; EgWRKY51: XP_010929316, *Elaeis guineensis*; HbWRKY17: XP_021671615, *Hevea brasiliensis*; JcWRKY17: XP_012076351, *Jatropha curcas*; LaWRKY11: AGZ01975, *Luffa aegyptiaca*; MeWRKY17: XP_021597536, *Manihot esculenta*; McWRKY17: XP_022141783, *Momordica charantia*; NnWRKY17: XP_010265415, *Nelumbo nucifera*; RcWRKY11: XP_002515353, *Ricinus communis*; TcWRKY17: XP_007011614, *Theobroma cacao*; VpWRKY11: AFV70622, *Vitis pseudoreticulata*; VvWRKY17: XP_002262775, *Vitis vinifera*; AtWRKY17: AT2G24570.1, *Arabidopsis*; AtWRKY11: At4G31550.1, *Arabidopsis*; ZmWRKY17: ACG39023.1, maize; GhWRKY17: ADW82098.1, cotton; CmWRKY17: AJF11725.1, *Chrysanthemum*. Conserved domain or motif was marked by the red line; NLS, nuclear localization signal. (**c**) Expression levels of *MfWRKY17* during dehydration treatment. Asterisks indicate significant difference (** *p* = 0.01) compared to the fully hydrated control (100% of initial weight).

Based on a sequence of a dehydration-inducible unigene (comp46861-c0-seq2) homologous to *AtWRKY17* [18], primers were designed to obtain an 999 bp open reading frame from cDNA of *M. flabellifolia* encoding a putative protein with 332 amino acid residues (Figure S1), designated *MfWRKY17* (GenBank accession no. MT383744). The calculated molecular weight of the deduced protein was 36.53 kDa. Multiple alignment of amino acid sequences with several most homologous sequences and several functionally-known WRKY17s from different plant species indicated that they contained a highly conserved WRKY domain, consisting of a WRKY motif and a C_2_H_2_ zinc-finger-like motif (Figure 1a). Therefore, MfWRKY17 could be classified into group II of the WRKY family. The conserved C-region, HARF motif, and a nuclear localization signal as those reported previously [19] were also found among these WRKY17s. However, the major parts of N-terminal and central regions were variable (Figure 1a). The subsequent phylogenetic analysis showed that MfWRKY17 was most homologous to VvWRKY17 of *Vitis vinifera* and VpWRKY11 of *Vitis pseudoreticulata* (Figure 1b). Compared with the functionally known WRKY17s, MfWRKY17 was phylogenetically closer with ZmWRKY17 [20] of maize than with AtWRKY17/11 of *Arabidopsis* [10], GhWRKY17 [21] of cotton, and CmWRKY17 [22] of *Chrysanthemum* (Figure 1b).

To verify expression pattern during dehydration, an expression analysis of *MfWRKY17* was performed. The results showed that transcript level of *MfWRKY17* exhibited a dehydration-inducible and fluctuated trend. It was significantly up-regulated at early dehydration (10% fresh weight loss). Although the expression level was decreased slightly at 25% of fresh weight loss, it increased since then and was significantly higher at 50% of fresh weight loss compared to that of the fully-hydrated control (Figure 1c). These results consistent with that obtained by transcriptome sequencing [18], suggesting that the expression of *MfWRKY17* was responsive to dehydration.

### 2.2. Subcellular Localization of MfWRKY17

**Figure 2:**
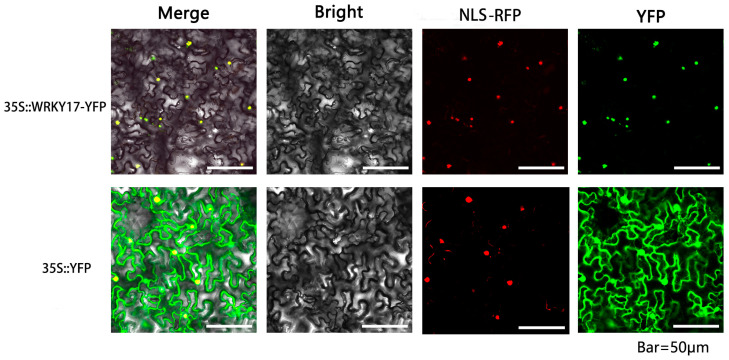
Subcellular localization of MfWRKY17. The constructs 35S::YFP and 35S::WRKY17-YFP were co-expressed with the nucleus marker NLS-RFP in *N. benthamiana* via *Agrobacterium*-mediated infiltration. YFP, yellow fluorescence protein; RFP, red fluorescence protein.

Using PSORTII, two nuclear localization signals (LSSSNKKRCHD and HCSKRRKNRV) were found within the MfWRKY17 protein (Figure S1). To verify this prediction, transient expression assays were conducted by expressing 35S: YFP and 2×35S:MfWRKY17-pHB-YFP fusion protein in *Nicotiana benthamiana* epidermal cells (Figure 2). The YFP signals of 35S::YFP control were distributed in various parts of the tobacco cell, while the yellow fluorescence of 2x35S:MfWRKY17-pHB-YFP protein was predominantly detected in nuclei.

### 2.3. Effects of MfWRKY17 Overexpression on Growth of Arabidopsis under Drought and Salt Stresses

To investigate potential roles of *MfWRKY17* in abiotic stress responses, *MfWRKY17* was introduced into *Arabidopsis* driven by CaMV 35S promoter. Kanamycin screening and PCR assays were used to identified positive transgenic lines. Two positive transgenic lines (homozygous T3 generation), Line-F and Line-M, were randomly chosen for further analysis. Through RT-PCR, it was found that expression of *MfWRKY17* was detectable in the two lines (Figure S2).

**Figure 3:**
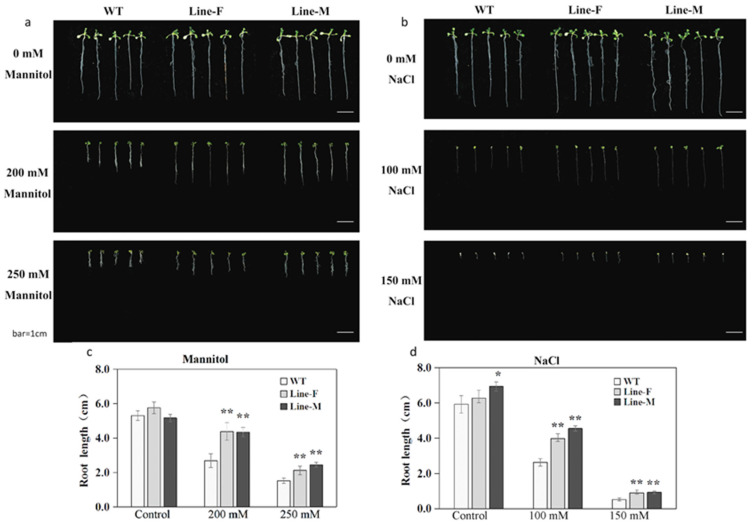
Growth of *MfWRKY17* overexpression seedlings under stress treatment. (**a**,**b**) showed morphology of seedlings of wild-type (WT) and transgenic lines growing on media, supplied with different concentrations of mannitol and NaCl, respectively. (**c**,**d**) indicated the measured root length of seedlings treated with mannitol and NaCl, respectively. Asterisks indicated significant difference (* *p* < 0.05, ** *p* < 0.01, by independent sample T-test) compared to WT.

The performance of seedlings of transgenic Line-F and Line-M, and WT control was monitored under salt and osmotic stresses. Under normal condition, the seedling growth of transgenic lines and WT was similar. However, under the presence of 200 mM and 250 mM mannitol, WT seedlings exhibited more severe growth inhibition than those of transgenic seedlings (Figure 3a). Similar results were also obtained in the presence of 100 and 150 mM NaCl (Figure 3b). The root length did not significantly differ between the transgenic lines and WT plants under non-stress conditions. However, under the treatments of 200 mM and 250 mM mannitol, and 100 mM and 150 mM NaCl, the *MfWRKY17* overexpression lines showed longer primary roots than those of WT control plants (Figure 3c,d).

**Figure 4:**
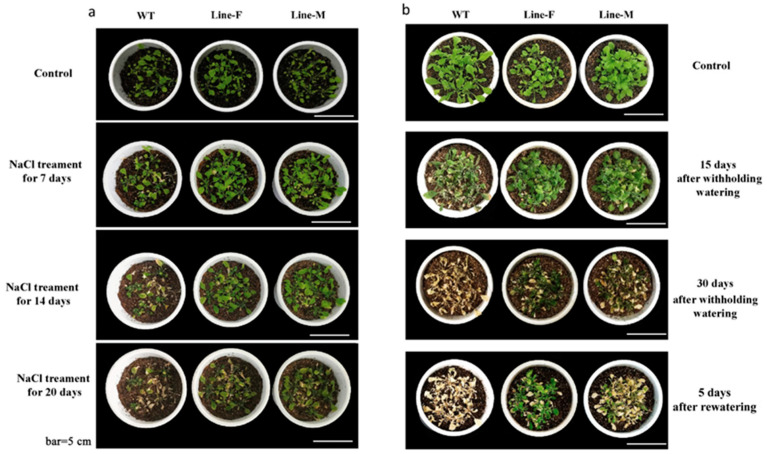
Morphological responses of wild-type (WT) and transgenic lines to salt (**a**) and drought treatments (**b**).

We further examined growth of transgenic lines and WT plants under long-term stress conditions in soil. Under normal conditions, WT and transgenic lines F and M grew well with similar phenotype. However, under the salt treatment, the WT plants wilted more severely than *MfWRKY17* overexpression lines (Figure 4a). Similarly, when withholding water for 30 days, most of the leaves of WT plants were completely wilted and withered. However, some plants of the transgenic lines survived and green leaves could be found. After rewatering for five days, most of WT plants did not recover to normal growth and died, whereas the *MfWRKY17* overexpression plants, especially Line-F, fully recovered (Figure 4b). 

### 2.4. Stomatal Closure and Water Loss Rates

**Figure 5:**
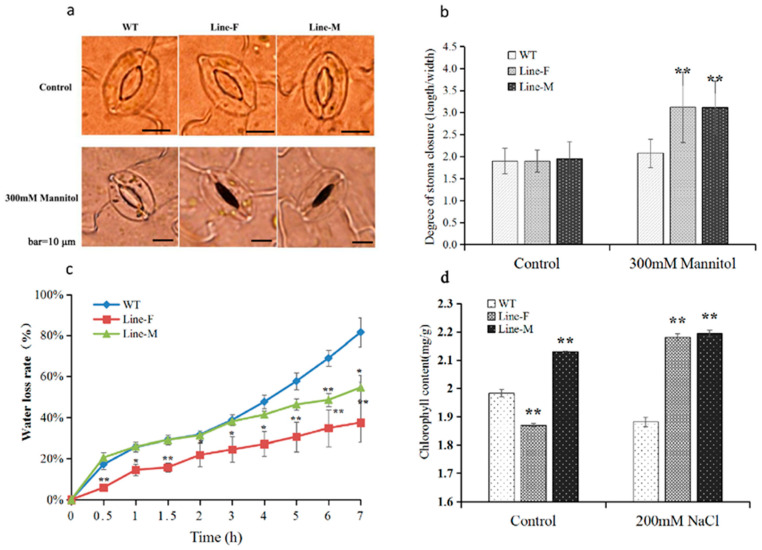
Physiological and biochemical effects of *MfWRKY17* overexpression on stress responses. (**a**,**b**), Stomatal closure in response to osmotic stress condition (300 mM mannitol); (**c**) Dynamic water loss rates of detached leaves; (**d**) Leaf chlorophyll contents under normal and salt treatments. Asterisks indicated significant difference (* *p* < 0.05, ** *p* < 0.01, by independent sample T-test) compared to WT.

We evaluated the abilities of water retention under stressful conditions. Under normal conditions, the stomatal aperture index (ratio of length/width of stomatal aperture) of WT plants was 1.8, and those of Line-F and Line-M plants were 1.8 and 1.9, respectively. After mannitol treatment, the stomatal aperture indices of Line-F and Line-M increased to 3.1 and 3.0, respectively, which were significant higher than that of WT (Figure 5a,b). Consistent with this result, Line-F showed significantly lower water loss rate than that of WT during the whole treatment process. Line-M showed similar water loss rate with WT at early stage of treatment. However, after 4 h, the water loss rate of WT increased faster than that of Line-M (Figure 5c).

### 2.5. Chlorophyll Content 

We evaluated chlorophyll content in the leaves of the transgenic lines and WT grown under normal conditions (control) or under salt stress (200 mM NaCl). Under normal growth conditions, chlorophyll content in leaves of WT plants was higher than that of Line-F and lower than Line-M. Under NaCl treatment, however, chlorophyll content of the transgenic lines was significantly higher than that of WT (Figure 5d).

### 2.6. Expression of Stress-Related Genes in MfWRKY17-Overexpressed *Arabidopsis* Plants

**Figure 6:**
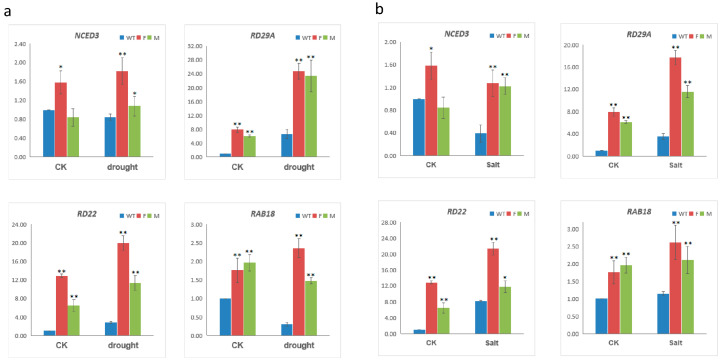
Expression levels of stress-responsive genes in wild-type (WT) and transgenic lines under drought (**a**) and salt (**b**) treatments. Y-axis showed the relative expression levels under normal (CK), drought, and salt treatments. *ACT2* gene was used as the internal control. Asterisks indicated significant difference (* *p* < 0.05, ** *p* < 0.01, by independent sample T-test) compared to WT.

To further investigate the role of *MfWRKY17* in response to salt and drought, the expression levels of some stress-related genes, including *NCED3*, *RD22*, *RD29A*, and *RAB18* that act as markers for monitoring salt or drought stress response pathways in *Arabidopsis*, were examined in WT and transgenic seedlings under both normal condition, NaCl and drought treatments. As shown in Figure 6, expression levels of almost all four genes in transgenic lines were higher than those of WT under normal condition. Under the drought stress, expression levels of *NCED3* increased in the transgenic lines, which were higher than that in WT. Expression levels of *RD29A* and *RD22* in WT and Line-F and Line-M increased under drought stress, however, the expression levels in overexpression lines were much higher than WT. For *RAB18*, its expression in Line-F significantly increased, whereas that in Line-M slightly decreased. However, expression levels in both lines still significantly higher than that in WT (Figure 6a). Similar trends were also found when the plants were treated by salt stress (Figure 6b). These results suggested that *MfWRKY17* may participate in response to salt and drought stresses by regulating expressions of stress-related genes in the abiotic stress response pathway.

## 3. Discussion

Although the involvement of WRKY TFs in biotic and abiotic stresses have been extensively studied, the knowledge of its roles mainly come from model plants, such as *Arabidopsis* and rice, and little is known about the role of WRKY TFs in non-model and stress-tolerant plants, such as the resurrection plant *M. flabellifolia*. In this study, we cloned a novel dehydration-induced group II of WRKY family member *MfWRKY17* from *M. flabellifolia*. In silico prediction and subcellular localization assay indicated that MfWRKY17 was localized to the cell nucleus, suggesting that *MfWRKY17* may function as a transcription factor (Figure S1, Figure 2).

We examined functions of *MfWRKY17* in response to abiotic stresses by overexpressing it in the model plant *Arabidopsis*. The transgenic lines exhibited longer roots on medium with osmotic and salt stresses (Figure 3). Moreover, adult plants also showed better growth under drought and salt treatments (Figure 4). These results indicated that the *MfWRKY17* overexpression could significantly enhance drought and salt tolerance in *Arabidopsis*, suggesting that it may positively regulate abiotic stress responses of *M. flabellifolia* to extremely arid environment.

In *Arabidopsis*, expression of *AtWRKY17* was induced by ABA, salt, and osmotic stress. Its knock-out mutant was exhibited slower germination and compromised root growth compared with wildtype [23]. However, more comprehensive studies suggested that WRKY17 usually acts as a negative regulator responsive to biotic or abiotic stresses. In *Arabidopsis*, WRKY17 functions as a negative regulator of basal resistance to *Pseudomonas syringae* pv *tomato* [19]. The constitutive expression of cotton *GhWRKY17* in *Nicotiana benthamiana* remarkably reduced plant tolerance to drought and salt stress [21]. *Chrysanthemum* CmWRKY17 acts as a transcriptional repressor, its overexpression increased the sensitivity of *Chrysanthemum* and *Arabidopsis* to salinity stress [22]. ZmWRKY17 may act as a negative regulator of the salt stress. Overexpression of *ZmWRKY17* in *Arabidopsis* significantly reduced plant tolerance to salt stress. Some stress-related genes in transgenic lines showed lower expression level than that in the WT under NaCl treatment [20]. However, our results suggest that MfWRKY17 of *M. flabellifolia* may function differently from the known WRKY17s. Structural comparison with other WRKY17s showed that the N-terminal and central regions are variable among them (Figure 1a). Previous study suggested that the N-terminal of ZmWRKY17 is essential for its interaction with other proteins [20]. Thus, the functional differences among WRKY17s may lie outside of the conserved domains. Further experimental evidences are needed to support this speculation.

Water loss rate is one of the most dependable physiological and phenotypic indicators used for assessing the plant water status under drought stress [24]. We found that the transgenic plants had lower water loss rate (Figure 5). Moreover, under osmotic stress condition, the degree of stomatal closure of transgenic plants were significantly higher than that of WT plants, suggesting that transgenic lines could regulate transpiration more efficiently through controlling stomatal closure. These results indicate that overexpression of *MfWRKY17* increases the ability for water retention under stresses.

Chlorophyll is one of the key components in photosynthesis and sensitive to abiotic stress [25]. In our study, overexpression of *MfWRKY17* results in NaCl-treated transgenic lines with significantly higher content of chlorophyll, which might be associated with the high photosynthetic activity (Figure 5). This might be one of the reasons for improved performance of the transgenic plants under salt stress.

Extensive studies have proved that WRKY TFs could positively regulate expression of stress-responsive related genes [26,27]. However, the negative regulatory roles of *WRKY17s* in gene expression were reported in other plant species. For example, overexpression of *Chrysanthemum* WRKY gene *CmWRKY17* inhibited expression of *AtRD29A*, *AtDREB2B*, as well as *AtSOS1-3* under salinity stress [22]. Overexpression of *ZmWRKY17* in *Arabidopsis* lowered the expression levels of *RD29A/B*, *RAB18*, *DREB1F*, and *NCED* under salt stress [20]. By contrast, in this study, overexpression of *MfWRKY17* in *Arabidopsis* upregulated expression levels of several stress-responsive genes under drought and salt stresses, i.e., *NECD3*, *RD22*, *RD29A*, and *RAB18* (Figure 6). 

*NCED3* encodes a key enzyme participating in ABA biosynthesis and is involved in the drought-stress response [28,29,30]. Under drought conditions, *GmWRKY16* transgenic plants showed higher levels of *NCED3* than that in WT plants as a result of ABA accumulation [27]. Similar results were obtained from other WRKY genes, such as *AtWRKY57* [26]. Therefore, the increased expression level of *NCED3* suggests that *MfWRKY17* may enhance drought and salt tolerance through altering ABA biosynthesis. This speculation is also supported by that ABA-responsive gene *RAB18* exhibited significantly higher expression levels in transgenic lines than those in WT both under untreated or stress-treated conditions (Figure 6). We also noticed that two stress-inducible marker genes, *Rd29A* and *Rd22*, showed significantly higher expression levels under drought and salt stresses. These results also suggested that *MfWRKY17* may act as a positive regulator for controlling of these stress- genes in the abiotic stress response pathway.

## 4. Materials and Methods

### 4.1. Plant Materials and Growth Conditions

Wild-type (WT) of *Arabidopsis* (Col-0) and transgenic plants (homozygous T3 generation) were grown in quartz sand at 25 °C (day)/20 °C (night), under the photoperiod of 16 h (day)/8 h (night) with approximately 100 µM photons m^−2^ s^−1^, and 60% relative humidity. All plant materials were irrigated once per week with Hoagland’s nutrient solution.

### 4.2. Cloning of MfWRKY17 and Sequence Analysis

Total RNAs were extracted from *M. flabellifolia* leaves using total RNA isolation system (LANBO, Chengdu, China). The reverse transcription reactions were performed employing the cDNA synthesis kit (TaKaRa, Dalian, China). The open reading frame sequence of *MfWRKY17* was obtained by reverse transcription polymerase chain reaction (RT-PCR) with specific primers (forward: 5′- TCCCCCGGGATGGCGGTTGAC -TTGCTC -3′ (*Sma*I site was underlined) and (reverse 5′- GACTAGTTCATGGTGTTGACTCAAACA -3′) (*Spe*I site was underlined). Subsequently, PCR product was ligated into the pEasy-T1 Simple vector (TRANSGENE, Beijing, China) and the positive clones were selected by PCR and then sequenced. The multiple sequence alignment was performed by BioEdit (version 7.0.5.5) [31], and the phylogenetic analysis was performed by MEGA (version 7.0) software [32]. The sequences used were those highly homologous to MfWRKY17, which were obtained by Blastp search against NCBI (https://www.ncbi.nlm.nih.gov/) nr protein dataset.

### 4.3. Subcellular Localization Assay of MfWRKY17

The complete coding region of *MfWRKY17* was cloned using the following primers: forward: 5′- ACCAGTCTCTCTCTCAAGCTTATGGCGGTTGACTTGCTC -3′ (*Hind*III site was underlined) and reverse 5′- GCTCACCATACTAGTGGATCCTG -GTGTTGACTCAAACACC -3′ (*BamH*I site was underlined), and then inserted into the pHB-YFP binary vector between the 2X35S promoter and *YFP* gene. The 2X35S:*MfWRKY17*-pHB-YFP (recombinant plasmids) and PHB-YFP alone were transformed into *Agrobacterium tumefaciens* strain GV3103, respectively. The NSL fused with RFP (red fluorescent protein) was co-expressed as a nuclecus marker [33]. *A. tumefaciens* with different constructs was used to infect *Nicotiana benthamiana* epidermal cells. The transformed *Nicotiana benthamiana* cells were then observed under a confocal microscope (Nikon A1, Tokyo, Japan). The experiment was repeated at least three times.

### 4.4. Plasmid Construction and Generation of MfWRKY17 Transgenic Arabidopsis

The coding sequence of *MfWRKY17* was amplified by PCR using a pair of primers containing *Sma*I and *Spe*I restriction sites. The *Sma*I and *Spe*I digested amplicons were purified and inserted into pGSA1403 driven by the CaMV (Cauliflower Mosaic virus) 35S promoter [34]. The recombinant vector 35S:*MfWRKY17*-pGSA1403 was transferred into the *A. tumefaciens* strain LBA4404 by the liquid nitrogen freezing and thawing method. For *Arabidopsis* transformation, *A. tumefaciens* cells containing the 35S:*MfWRKY17*-pGSA1403 construct were transformed into *Arabidopsis* Col-0 plants by the floral dip method. T1 seeds were screened in 1/2 MS (Murashige and Skoog) culture medium containing 50 mg/L Kanamycin (Aokesw, Qingdao, China). Homozygous T3 lines were obtained by self-pollination. Two positive lines confirmed by RT-PCR were selected for further analysis.

### 4.5. Phenotype Analysis under Osmotic, Drought, and Salt Stresses

For osmotic and salt treatments, seeds of WT and *MfWRKY17*-overexpressing lines were surface sterilized and sown on 1/2 MS culture medium containing 3% sucrose and 0.7% agar. Seeds were grown in a growth chamber at 23 °C under long-term illumination (16 h/8 h, light/dark) for four days. Then, the seedlings were transferred onto 1/2 MS culture medium with different concentrations of mannitol (0–250 mM) and NaCl (0–150 mM), respectively. Seven days after vertical growth in the medium, the root lengths of the seedlings were measured.

For survival evaluation, seeds of *MfWRKY17* transgenic plants and WT were planted in 7 cm-diameter pots filled with peat soil. Four-week-old plants were exposed to drought and salt stress by withholding water for 21 days and irrigating with 300 mM NaCl for 14 days, respectively. After three week of drought stress treatment, the plants were rewatered and allowed to recover from the drought stress conditions. 

### 4.6. Water Loss Rate

To measure plant transpiration (water loss), rosette leaves of four-week-old transgenic lines and WT were sampled and weighed, and placed on a weighing paper at 25 ℃, 60% relative humidity to dry. The weight of the leaf samples were measured at 0.5 h, 1 h, 1.5 h, 2 h, 3 h, 4 h, 5 h, 6 h, and 7 h. Three replicates were measured for each line.

### 4.7. Stomatal Aperture Analysis

Ten leaves with similar size were sampled from WT and transgenic plants, respectively. Rosette leaves were floated in 100 mL MES-KCl solutions (pH = 6.15) (50 mM KCl, 0.1 mM CaCl_2_, 10 mM MES) with additional 300 mM mannitol, and exposed to light for 2.5 h. An optical microscope (Olympus ix71, Tokyo, Japan) was used to observe the stomata on epidermal strips. The width and length of stomatal pores were measured by Image J (http://rsbweb.nih.gov/ij), and then were used to calculate ratio of width to length. The significance of difference between WT and transgenic plants was assessed by Student’s t-test.

### 4.8. Chlorophyll Content 

Four-week-old WT and transgenic plants were exposed to salt stress by irrigating with 200 mM NaCl solutions for two days. Chlorophyll in 0.5 g fresh leaves per sample was extracted with 95% alcohol. Chlorophyll content was assayed by measuring absorbance at 649 nm and 665 nm with a spectrophotometer. 95% ethanol was used as a blank control. The assays were performed three times with three biological repetitions.

### 4.9. Reverse Transcription PCR (RT-PCR) and Quantitative Real-Time PCR (qRT-PCR)

To detect if MfWRKY17 expresses in the transgenic lines, the total RNAs were extracted from WT and transgenic lines using the RNAout Kit (TIANGEN, Beijing, China) and treated with DNaseI (RNase free). The first-strand cDNA was synthesized using the PrimeScript™ 1st Strand cDNA Synthesis Kit (Takara, Dalian, China). The RT-PCR amplifications were performed using the primers as same as those used for cloning.

Dehydration treatment of *M. flabellifolia* was performed according to Ma et al. (2015) with three replicates [18]. The samples in 100%, 90%, 75%, and 50% of initial weight were used for expression analysis. To determine the expression patterns of stress-responsive genes in transgenic line, seeds of *MfWRKY17* transgenic *Arabidopsis* and WT were planted in 7 cm-diameter pots filled with peat soil. Four-week-old plants were exposed to drought and salt stress. Leaves with similar size and developmental stages were sampled after withholding water for 36 h or irrigating with 200mM NaCl for 24 h, respectively. The samples were frozen in liquid nitrogen and stored in −80 ℃ refrigerator immediately. qRT-PCR was performed using the SYBR^®^ Premix Ex Taq™ (Tli RNaseH) kit (Takara, Dalian, China) with a CFX96 Real-Time PCR machine (Bio-Rad, Hercules, CA, USA). Each reaction contained 10 µL of SYBR Green Master mix, 0.8uL of the primers (10 uM) and 2 µL of cDNA in a final volume of 20 µL. The PCR was performed using the following parameters: 95 ℃ for 10min; and 40 cycles of 95 ℃ for 10 s, 58 ℃ for 20 s and 72 ℃ for 20 s. The primers used for qRT-PCR are listed in Table S1. The *ACT2* (*actin-2*, locus name At3g18780) gene of *Arabidopsis* was used as the reference. The relative expression levels were determined using 2^−ΔΔ*C*T^ method. The qRT-PCR was performed with at least three technical and three biological repeats.

### 4.10. Statistical Analyses

Statistical analyses were performed using SIGMAPLOT 12.5 and SPSS (version 26.0, IBM, Armonk, NY, USA). ** *p* < 0.01 and * *p* < 0.05 represent significant differences compared to the control.

## 5. Conclusions

The present study reported characterization of a dehydration-responsive WRKY transcription factor *MfWRKY17* of *M. flabellifolia*. Unlike known *WRKY17s* in other plant species acting as negative regulator in biotic or abiotic stress responses, overexpression of *MfWRKY17* in *Arabidopsis* significantly increased osmotic, drought and salt tolerances. Further investigation indicated that *MfWRKY17* may participate in increasing water retention, maintaining chlorophyll content, regulating ABA biosynthesis and stress-related gene expression. These results suggest that *MfWRKY17* may function as a positive regulator of stress tolerance in the resurrection plant *M. flabellifolia*.

## Supplementary Materials

Supplementary materials can be found at https://www.mdpi.com/1422-0067/21/13/4603/s1.
