# Supplementary material for: Heterologous Expression of Dehydration-Inducible MfWRKY17 of Myrothamnus Flabellifolia Confers Drought and Salt Tolerance in Arabidopsis
Source: Int J Mol Sci. 2020 Jun 29;21(13):4603. doi: 10.3390/ijms21134603 (PMC7370056; doi:10.3390/ijms21134603)
Supplement: Supplementary file 1 [file ijms-21-04603-s001.pdf]

```
1      ATGGCGGTTGACTTGCTCGGGTATACAAAGATGGACGAACAGATGGCGATCCAAGAGGCCGCTTCAGCTGGATTAAGGAGCATGGAGCAT
1       M  A  V  D  L  L  G  Y  T  K  M  D  E  Q  M  A  I  Q  E  A  A  S  A  G  L  R  S  M  E  H

91     CTGATCCTTGTATTATCACATCAGTCACACCAATCTAACCAATTAGATTGCAAAGAAATCACAGATTTCACCGTCTCAAAGTTCAAAAAG
31      L  I  L  V  L  S  H  Q  S  H  Q  S  N  Q  L  D  C  K  E  I  T  D  F  T  V  S  K  F  K  K

181    GTCATCTCCATCTTAAATCGAACAGGTCATGCTCGATTTCGCCGTGGTCCTTCTCAATCATCGGCTTCTACGTCTGTTCCTCAATCGCAG
61      V  I  S  I  L  N  R  T  G  H  A  R  F  R  R  G  P  S  Q  S  S  A  S  T  S  V  P  Q  S  Q

271    ACGTTCAATCTAACTTCGACCGCCTTCGTACAATCTAAGCCACGGCCGCAGCAGCAGCCACAGCCGCAGCCACAGCCACAATATCTAACG
91      T  F  N  L  T  S  T  A  F  V  Q  S  K  P  R  P  Q  Q  Q  P  Q  P  Q  P  Q  P  Q  Y  L  T

361    CTCGACTTCACAAAACCTAATATGCTGTCTTCAAACTGTGAGCAGCTTACTGACGTAGTTTCGACGAGTCAATTCACAAAGGAGAGCGTT
121     L  D  F  T  K  P  N  M  L  S  S  N  C  E  Q  L  T  D  V  V  S  T  S  Q  F  T  K  E  S  V

451    AGCATATCGCAGCCTATGTCTTCGACAAACTCGTCCTTCATGTCGTCCATCACCGAAAACGGAAGTGTTTCAGACGGAAAACAAGGGTCG
151     S  I  S  Q  P  M  S  S  T  N  S  S  F  M  S  S  I  T  E  N  G  S  V  S  D  G  K  Q  G  S

541    TCTTTGTTCTTAGCTCCGGCGCCGGCAGTTTCCGCCGGTAAACCACCTTTATCGTCGTCCAATAAGAAGAGATGTCATGATCATGATCAT
181     S  L  F  L  A  P  A  P  A  V  S  A  G  K  P  P  L  S  S  S  N  K  K  R  C  H  D  H  D  H

631    GATCATTCCGATGAACTTTCCGGGAAGCAATCCAGTTCCGGCCGATGTCACTGCTCGAAAAGAAGGAAAAATCGGGTAAAGAGTACGATT
211     D  H  S  D  E  L  S  G  K  Q  S  S  S  G  R  C  H  C  S  K  R  R  K  N  R  V  K  S  T  I

721    AGAGTGCCGGCAATTAGTTCAAAAATAGCCGATATTCCGCCAGACGAATATTCTTGGAGAAAGTACGGACAAAAGCCGATCAAGGGTTCG
241     R  V  P  A  I  S  S  K  I  A  D  I  P  P  D  E  Y  S  W  R  K  Y  G  Q  K  P  I  K  G  S

811    CCCTACCCAAGGGGCTATTATAAATGCAGTAGCTTAAGGGGCTGTCCTGCGAGAAAACACGTCGAGCGTGCTCCAGATGATCCAACGATG
271     P  Y  P  R  G  Y  Y  K  C  S  S  L  R  G  C  P  A  R  K  H  V  E  R  A  P  D  D  P  T  M

901    TTGATCGTCACCTATGAAGGGGAGCACCGGCACTCAAAACTCACATCGCAGGAGAATATTTCTGGAGGTGCAGGTTTGGTGTTTGAGTCA
301     L  I  V  T  Y  E  G  E  H  R  H  S  K  L  T  S  Q  E  N  I  S  G  G  A  G  L  V  F  E  S

991    ACACCATGA
331     T  P  *
```

Fig. S1 Nucleotide sequence and deduced amino acid sequence of coding region of *MfWRKY17*. Star indicated stop codons. The predicted nuclear localization signals were underlined.

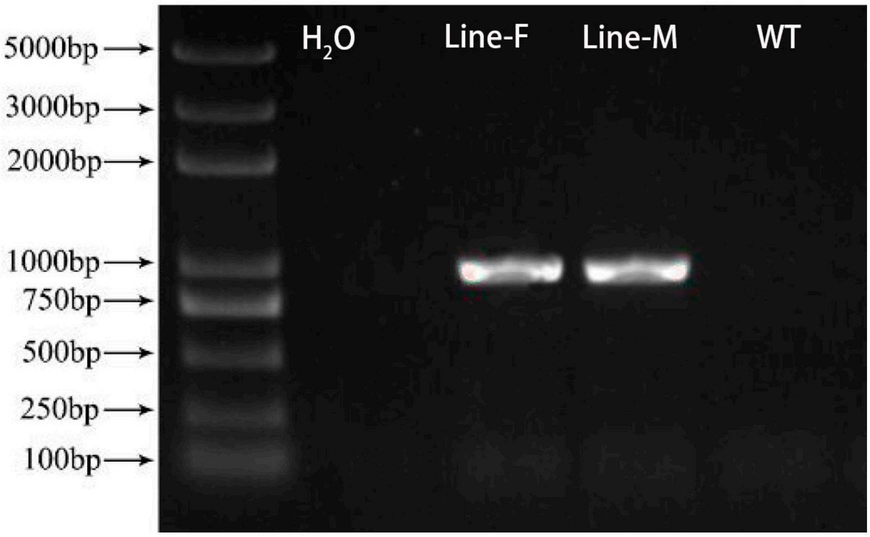

Fig. S2 Molecular detection of *MfWRKY17* in transgenic lines by RT-PCR. WT, wild type. By using the cDNA extracted from leaf of WT and transgenic lines as templates, the complete coding sequences of *MfWRKY17* was amplified.

Table S1 Primers used for qRT-PCR assays.

Gene	primer 5’-3’	
	forward	reverse
Actin	GGAAGGATCTGTACGGTAAC	TGTGAACGATTCCTGGACCT
MfWRKY17	TCCTCAATCGCAGACGTTCA	CTGCGATATGCTAACGCTCTC
NCED3	CGAGCCGTGGCCTAAAGTCT	GCTCCGATGAATGTACCGTGA
RD22	FACTTGGTAAATATCACGTCAGGGCT	CTGAGGTGTTCTTGTGGCATACC
RD29A	GATAACGTTGGAGGAAGAGTCGG	TCCTGATTCACCTGGAAATTTCG
RAB18	GCAGTATGACGAGTACGGAAATCC	CCTTGTCCATCATCCGAGCTAGA
